# A population study of screening history and diagnostic outcomes of women with invasive cervical cancer

**DOI:** 10.1002/cam4.3951

**Published:** 2021-05-21

**Authors:** Vicki B. Benard, J. Elizabeth Jackson, April Greek, Virginia Senkomago, Warner K. Huh, Cheryll C. Thomas, Lisa C. Richardson

**Affiliations:** ^1^ Division of Cancer Prevention and Control Centers for Disease Control and Prevention Atlanta Georgia USA; ^2^ Battelle Seattle Washington USA; ^3^ University of Alabama at Birmingham Birmingham Alabama USA

**Keywords:** cancer registries, cervical cancer screening, cervical cancer survivors

## Abstract

**Background:**

Despite advances to prevent and detect cervical cancer, national targets for screening have not been met in the United States. Previous studies suggested that approximately half of women who developed cervical cancer were not adequately screened. This study aimed to provide an updated examination of women's screening and diagnostic practices five years prior to an invasive cervical cancer diagnosis.

**Methods:**

The study included women age 21 years and older diagnosed with invasive cervical cancer in 2013–2016 from three population‐based state cancer registries in the United States. Medical records abstraction identified screening history and diagnostic follow‐up. A mailed survey provided sociodemographic data. Screening was a Pap or human papillomavirus (HPV) test between 6 months and 5 years before diagnosis. Adequate follow‐up was defined per management guidelines.

**Results:**

Of the 376 women, 60% (n = 228) had not been screened. Among women who received an abnormal screening result (n = 122), 67% (n = 82) had adequate follow‐up. Predictors of: (a) being screened were younger age, having a higher income, and having insurance; (b) adequate follow‐up were having a higher income, and (c) stage 1 cervical cancer were being screened and younger age.

**Conclusion:**

Unlike other cancer patterns of care studies, this study uses data obtained from medical records supplemented with self‐report information to understand a woman's path to diagnosis, her follow‐up care, and the stage of her cervical cancer diagnosis. This study provides findings that could be used to reach more unscreened or under screened women and to continue lowering cervical cancer incidence in the United States.

## Graphical abstract

An important risk factor for cervical cancer is not being screened. This study found that 60% of enrolled cervical cancer survivors did not receive appropriate screening in the five years prior to their diagnoses. Increasing screening in rarely and never‐screened women as well as timely follow‐up is crucial to continuing to lower cervical cancer incidence in the U.S.

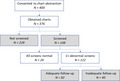

## 1 INTRODUCTION

Cervical cancer is largely preventable through screening that allows for the detection and treatment of cervical cancer precursors.^1^ With screening programs that were introduced in the 1950 s, cervical cancer incidence and death rates have decreased drastically over time.^2^ However, each year approximately 13,000 women are diagnosed with and more than 4,000 women die from cervical cancer,^3^ and cervical cancer disproportionally affects women who are Hispanic, Black, or from low socioeconomic backgrounds.^4^ Previous studies have suggested that approximately half of the women who developed cervical cancer in the United States were not adequately screened.^5^, ^6^ A new statewide population‐based study conducted in New Mexico found that 64% of the women with cervical cancer had not been screened.^7^ When factors such as poor access to care interfere with screening or appropriate follow‐up, women may be diagnosed at later stages and have lower survival.^1^, ^7^, ^8^ To reduce cervical cancer burden and reach national objectives including reducing incidence rates, death rates, and health disparities, it is essential that we understand the risk factors for non‐screening and inadequate follow‐up care.

The Centers for Disease Control and Prevention (CDC) Case Investigation of Cervical Cancer (CICC) Study took a unique approach to reconstruct the time before a woman's cervical cancer diagnosis, combining her screening history with diagnosis information and sociodemographic factors to understand her prognosis and outcomes.^4^ This paper aims to answer the following questions: (a) What proportion of cervical cancer survivors were screened during the five years prior to diagnosis? What factors predicted screening? (b) What proportion of survivors received the recommended follow‐up for their first abnormal test in a timely manner? What factors predicted adequate follow‐up? and (c) What factors were associated with diagnosis at an early stage?

## 2 METHODS

The study included all cervical cancer survivors diagnosed with invasive cervical cancer 21 years and older in three United States state cancer registries^9^; those diagnosed between 2014 and 2016 in Michigan and New Jersey, and between 2013 and 2016 in Louisiana. Details of the study are reported elsewhere.^4^ Study packets were mailed containing questionnaires and requests for consent to collect data from medical records. Of the 1,730 eligible women, 28% (n = 481) enrolled in the study and 23% (n = 400) consented to chart abstraction. Additional information on response rate has been reported.^4^ Of the women who consented, charts were available for 376 (94%). Abstractors were asked to evaluate the completeness of the data they were able to obtain for each woman based on requests made and the responses. Abstractors judged they had obtained all relevant records for 251 women (67%), and at least some relevant records for 125 (33%).

Women's medical chart and survey data were combined with data provided directly by the registries. Trained abstractors collected medical record data for the 5‐year period up to the date of invasive cervical cancer diagnosis collecting the date, procedure or test type(s), and diagnoses for each screening and diagnostic procedure. The survey collected data on household income, insurance status, and race/ethnicity. Finally, registries provided data on cancer histology, cancer staging, and date of diagnosis. Variables not included in analyses because they were not significant predictors of screening (*p *< .05) were marital status, metropolitan residence and a census track poverty indicator (source: registries), and other cancer diagnoses or cervical procedures more than 5 years prior (source: medical records).

### 2.1 Dependent variables

#### 2.1.1 *Screening status (n* =* 376)*

**FIGURE 1:**
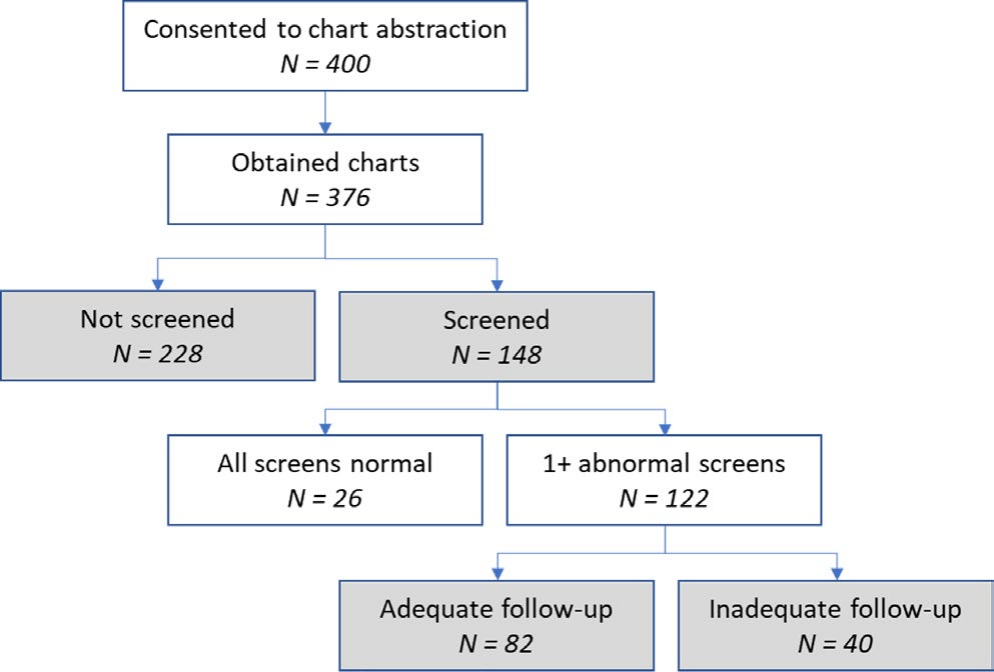
Study participants in the Case Investigation of Cervical Cancer (CICC) Study by screening and follow‐up

Cervical cancer screening recommendations at the time of the study recommended average‐risk women ages 21–65 receive a Pap test every 3 years, or for women ages 30–65 every 5 years if accompanied by HPV testing (co‐testing).^10^ In this study, the dates in the medical records were used to classify women as “screened for cervical cancer” if they had a Pap test (with or without an HPV test) 6 months to 5 years before the date of diagnosis. This 6‐month cutoff is a timeframe used by similar studies,^5^, ^11^ and assumes that Pap tests within 6 months would most likely represent follow‐up testing to confirm abnormal results rather than screening.^10^ Women were classified as unscreened if she had no screening in the 5‐year timeframe or if she had a Pap test less than 6 months from her diagnosis. Women were classified by their screening status (Figure 1): not screened (n = 228) versus screened (n = 148), with screened women grouped according to screening results and follow‐up. Unscreened women had their results reported in tables (Table 3).

Cytology results were reported in the Bethesda System terminology.^12^ Co‐testing was defined as an HPV test within 7 days. HPV test results were reported as negative or positive for the detection of high‐risk HPV DNA.

#### 2.1.2 *Adequate follow*‐*up (n *= *122)*

We evaluated adequacy of follow‐up of the first abnormal Pap or positive HPV test recorded in the study period among screened women with at least one abnormal test (this analysis was not performed for unscreened with test results); an abnormal test result was anything requiring, at a minimum, a shortened screening interval per management guidelines.^10^ The algorithm utilized for assigning follow‐up adequacy is provided in Table 1; assignment to adequate versus inadequate follow‐up was based on national guidelines. In cases where guidelines provide a range, we used the upper bound.

**TABLE 1:** Method for Determining Adequacy of Follow‐up and Follow‐up Group

Cervical cancer screening results	Reasonable minimum follow‐up^a^	Follow‐up group
Unsatisfactory cytology:		
HPV unknown or negative	Repeat cytology in 4 months	Repeat test
HPV positive	Repeat cytology in 4 months or colposcopy	Repeat test
Negative cytology and HPV+:		
Absent/insufficient TZ	Co‐testing within 1 year	Repeat test
Not genotyped or 16/18‐	Co‐testing within 1 year	Repeat test
HPV genotype 16/18+	Colposcopy within 3 months	Biopsy
ASC‐US:		
HPV unknown	Repeat cytology within 1 year	Repeat test
HPV negative	Repeat co‐testing within 3 years	Repeat test
HPV positive	Colposcopy within 3 months	Biopsy
LSIL:		
HPV unknown	Colposcopy within 3 months	Biopsy
HPV negative	Repeat co‐testing within 1 year or colposcopy	Repeat test
HPV positive	Colposcopy within 3 months	Biopsy
ASC‐H	Colposcopy in 2 months	Biopsy+^b^
HSIL	Colposcopy in 2 months or excisional procedure in 3 months	Biopsy+^b^
ACG or adenocarcinoma in situ	Colposcopy in 2 months or excisional procedure in 3 months	Biopsy+^b^
Squamous cell carcinoma	Colposcopy in 1 month or excisional procedure in 2 months	Biopsy+^b^

Abbreviations: ‐, negative; +, positive; AGC, atypical glandular cells; ASC‐H, atypical squamous cells cannot rule out high‐grade; ASC‐US, atypical squamous cells of undetermined significance; HPV, human papillomavirus; HSIL, high‐grade intraepithelial lesion; LSIL, low‐grade squamous intraepithelial lesion; TZ, transformation zone component.

^a^ Receipt of a more extensive procedure in the same timeframe was considered reasonable follow‐up.

^b^ Biopsy+: Possible high‐grade finding and/or excisional procedure recommended.

#### 2.1.3 *Cancer staging (n* =* 349)*

Women for whom cancer staging was reported (93%) were classified according to whether their cancer was diagnosed at Stage I versus Stages II–IV. The stage at diagnosis was defined according to the AJCC TNM classification system.

### 2.2 Independent Variables

#### 2.2.1 Sociodemographics

Age at diagnosis was estimated from month and year of birth recorded in the medical record and diagnosis date; women were grouped by age: 23–39; 40–54; 55 years and older. Women's race/ethnicity (“What is your race or racial heritage?”; “Are you of Hispanic or Latina origin?”) was classified as non‐Hispanic White or other races/ethnicities combined. For five women who did not report race/ethnicity, we substituted race/ethnicity from their medical records. Household income (“At the time of your cervical cancer diagnosis, which of the following categories best described your annual income?”) was grouped: less than $30,000; $30,000–89,999; $90,000 and above. Insurance status during the study period (“During the five years prior to your cervical cancer diagnosis, were you covered by health insurance that paid for all or part of your medical care?”) was coded yes/no.

#### 2.2.2 Clinical and screening history

Tubal ligation prior to diagnosis (yes/no) was collected from medical records. The number of overall and abnormal Pap and HPV tests performed during the study period were calculated from medical records data.

#### 2.2.3 Diagnostic findings

Cervical cancer histology was reported by registries (squamous cell carcinoma, adenocarcinoma, and other cancer). To evaluate the impact of abnormal screenings on adequate follow‐up, the first abnormal Pap or positive HPV test recorded in the study period among women with an abnormal test was grouped in three categories according to recommended follow‐up: repeat testing or optional colposcopy (“repeat testing”); colposcopy within 3 months (“biopsy”); possible high grade finding (i.e., ASC‐H or more severe) and/or excisional procedure recommended (“biopsy+”). This classification strategy was intended to identify potential differences in following the management guidelines where co‐testing has been introduced. Recommended follow‐up group for screened and unscreened women is provided in Table 1.

### 2.3 Statistical Analysis

Analyses were completed using Stata statistical software (version 15.1; StataCorp, College Station, Texas). Distribution of independent variables and diagnostic outcomes are presented stratified by screening outcome group. Logistic regression was used to test associations between selected independent and dependent variables. Variables significant in unadjusted models were included in adjusted models. Multiple imputation of missing data was conducted for use in logistic regression models for all three outcomes. Multiple imputation by chained equations included all variables present in the respective unadjusted logistic regression models, and was applied in unadjusted and adjusted models for each outcome separately.^13^ Significance level was set at *p* < 0.05.

This study was approved by the CDC’s Human Research Review Committee and by each cancer registry's institutional review board. Study data were collected and managed using REDCap electronic data capture tools hosted at Vanderbilt University.^14^

## 3 RESULTS

Of the 376 women in this study, 228 (60%) were not screened in the 5‐year study period. Among unscreened women, 72 (32%) had no tests done, 156 (68%) had 1 or 2 Pap and/or HPV tests within 6 months of their diagnosis, and 93 (41%) had at least one HPV test within 6 months of diagnosis. Of the 148 (40%) women defined as screened, all received at least one Pap test and 106 (72%) received at least one HPV test; only 4 HPV tests were collected without a Pap test on the same day.

Sociodemographic and clinical characteristics of the women in the study by screening status and follow‐up are provided in Table 2. Overall, 30% of women diagnosed with cancer were younger than 40 at diagnosis and 34% were races/ethnicities other than non‐Hispanic White. Nearly half (43%) were in the lowest income group (<$30,000) and 26% were uninsured. Among unscreened women, about half (51%) were in the lowest income group and 34% were uninsured. Overall, 27% of women had tubal ligation, compared with 33% of unscreened women. Half of women overall (51%) and 43% of unscreened women were diagnosed at Stage I.

**TABLE 2:** Characteristics of Women in the Case Investigation of Cervical Cancer (CICC) Study by screening and follow‐up status^a^

	Overall *(n=376)*	Screening Status		Follow‐up Among Screened (n=148)		
		Not Screened *(n=228)*	Screened *(n=148)*	All Screens Normal *(n=26)*	Adequate Follow‐up *(n=82)*	Inadequate Follow‐up *(n=40)*
*Socio‐demographics*						
Age at diagnosis, y						
23–39	30.0	20.6	44.6	30.8	43.9	55.0
40–54	41.5	44.3	37.2	42.3	35.4	37.5
≥55	28.5	35.1	18.2	26.9	20.7	7.5
Race/Ethnicity:						
Non‐Hispanic White	66.2	61.0	74.3	84.6	74.4	67.5
Other	33.8	39.0	25.7	15.4	25.6	32.5
Income (annual):						
< $30,000	43.4	51.3	31.1	23.1	24.4	50.0
$30,000 – $89,999	30.3	28.9	32.4	38.5	32.9	27.5
≥ $90,000	17.8	9.2	31.1	30.8	37.8	17.5
Missing	8.5	10.5	5.4	7.7	4.9	5.0
Insured during study period:						
Not insured	26.2	33.8	14.5	4.0	11.1	28.2
Insured	73.8	66.2	85.5	96.0	88.9	71.8
*Clinical and Screening History*						
Tubal ligation						
Yes	26.9	33.3	16.9	7.7	20.7	15.0
No	73.1	67.7	83.1	92.3	79.3	85.0
At least one Pap	80.6	68.0	100.0	100.0	100.0	100.0
Mean (SD) Paps^b^	2.0 (1.6)	1.0 (0.2)	3.1 (1.7)	2.2 (1.1)	3.5 (1.8)	2.8 (1.6)
Mean (SD) abnormal^b^	1.2 (0.9)	1.0 (0.3)	1.4 (1.2)	0.1 (0.3)^c^	1.6 (1.0)	1.7 (1.3)
At least one HPV test^d^	52.9	40.8	71.6	38.5	72.0	92.5
Mean (SD) tests^b^	1.7 (1.4)	1.0 (0.2)	2.2 (1.7)	1.5 (0.7)	2.6 (1.8)	1.8 (1.4)
Mean (SD) positive^b^	1.4 (1.3)	0.9 (0.4)	1.8 (1.6)	0.3 (0.5)^c^	2.1 (1.8)	1.7 (1.4)
*Invasive Cancer Diagnosis*						
Histology:						
Squamous cell carcinoma	70.0	78.1	57.4	46.2	57.3	65.0
Adenocarcinoma	27.7	20.6	38.5	42.3	41.5	30.0
Other cancer	2.4	1.3	4.1	11.5	1.2	5.0
Staging:						
Stage I	51.3	43.4	63.5	65.4	62.2	65.0
Stage II	18.9	24.1	10.8	7.7	14.6	5.0
Stage III	17.0	19.7	12.8	15.4	13.4	10.0
Stage IV	5.6	6.1	4.7	3.9	3.7	7.5
Not reported	7.2	6.6	8.1	7.7	6.1	12.5

^a^ Column percentages unless otherwise indicated.

^b^ Among women with at least one Pap or HPV test performed, respectively.

^c^ For these screened women, their only abnormal Pap/HPV test was collected on the day of diagnosis.

^d^ All but 4 HPV tests total were collected at the same time as a Pap test.

Table 3 provides test results for the first abnormal test for screened and unscreened women with at least one abnormal Pap or HPV test (n = 122 and n = 150, respectively), and for screened women by adequacy of follow‐up (adequate, n = 82; inadequate, n = 40). The most common findings among screened women overall were negative cytology with HPV positive (n = 27; 22%), ASC‐US with HPV positive (n = 21; 17%), and HSIL (n = 20; 16%). Among unscreened women, the most frequent findings were HSIL (n = 62; 41%), AGC (n = 33; 22%), and squamous cell carcinoma (n = 16; 11%). The most frequently recommended follow‐up based on their first abnormal test among women overall was a colposcopy or excisional procedure within 1–3 months (biopsy+) (62%); among unscreened women this was the recommended follow‐up for 81% versus for 39% of screened women.

**TABLE 3:** First abnormal screening test result and recommended follow‐up group by screening status and adequacy of follow‐up among screened

	Screened						Not Screened	
	Total		Adequate		Inadequate		Total	
	No.	(%)	No.	(%)	No.	(%)	No.	(%)
First abnormal screening test result								
Unsatisfactory cytology:								
HPV unknown or negative	3	(2.5)	2	(2.4)	1	(2.5)	2	(1.3)
HPV positive	0	(0.0)	0	(0.0)	0	(0.0)	3	(2.0)
Negative cytology and HPV+:								
Absent/insufficient TZ	1	(0.8)	0	(0.0)	1	(2.5)	0	(0.0)
Not genotyped or 16/18‐	18	(14.8)	13	(15.9)	5	(12.5)	0	(0.0)
HPV 16/18+	8	(6.6)	3	(3.7)	5	(12.5)	2	(1.3)
ASC‐US:								
HPV positive	21	(17.2)	12	(14.6)	9	(22.5)	10	(6.7)
HPV negative	4	(3.3)	4	(4.9)	0	(0.0)	4	(2.7)
HPV unknown	6	(4.9)	4	(4.9)	2	(5.0)	2	(1.3)
LSIL:								
HPV positive	7	(5.7)	2	(2.4)	5	(12.5)	5	(3.3)
HPV negative	1	(0.8)	1	(1.2)	0	(0.0)	0	(0.0)
HPV unknown	6	(4.9)	3	(3.7)	3	(7.5)	1	(0.7)
ASC‐H	11	(9.0)	10	(12.2)	1	(2.5)	10	(6.7)
HSIL	20	(16.4)	16	(19.5)	4	(10.0)	62	(41.3)
AGC or adenocarcinoma	15	(12.3)	11	(13.4)	4	(10.0)	33	(22.0)
Squamous cell carcinoma	1	(0.8)	1	(1.2)	0	(0.0)	16	(10.7)
Total	122	(100)	82	(100)	40	(100)	150	(100)
Recommended follow‐up group ^a^								
Repeat testing	33	(27.1)	24	(29.3)	9	(22.5)	11	(7.3)
Biopsy	42	(34.4)	20	(24.4)	22	(55.0)	18	(12.0)
Biopsy+ ^b^	47	(38.5)	38	(46.3)	9	(22.5)	121	(80.7)
Total	122	(100)	82	(100)	40	(100)	150	(100)

Abbreviations: ‐, negative; +, positive; AGC, atypical glandular cells; ASC‐H, atypical squamous cells cannot rule out high‐grade; ASC‐US, atypical squamous cells of undetermined significance; HPV, human papillomavirus; HSIL, high‐grade intraepithelial lesion; LSIL, low‐grade squamous intraepithelial lesion; TZ, transformation zone component.

^a^ Chi‐square *p* <.001 for relationship of screening status with follow‐up recommendation; chi‐square *p* =.003 for relationship of follow‐up adequacy with follow‐up recommendation among screened women.

^b^ Biopsy+: Possible high‐grade finding and/or excisional procedure recommended.

Table 4 provides the unadjusted and adjusted predictors for (a) screening status, (b) adequacy of follow‐up care among screened women, and (c) staging at diagnosis. Adjusted models showed there were lower odds of being screened among women age 40–54 years (adjusted OR [aOR] 0.43; 95% CI 0.25–0.73) or 55 years and older (aOR 0.26; 95% CI 0.14–0.47) compared to women under 40, and among women who had tubal ligation (aOR 0.55; 95% CI 0.31–0.96) compared to those who did not. Higher odds of being screened were found among women in the highest income group (aOR 3.62; 95% CI 1.76–7.43) compared to women in the lowest income group, and among women with insurance (aOR 2.09; 95% CI 1.15–3.81) compared to those without. Among screened women, higher odds of receiving adequate follow‐up care were found among women in the highest income group (aOR 3.96; 95% CI 1.24–12.64) compared with women in the lowest income group, and among women with the biopsy+follow‐up recommendation (aOR 5.02; 95% CI 1.85–13.62) compared to those with a more moderate recommendation for colposcopy within 3 months. Lower odds of being diagnosed at Stage 1 compared to higher Stages (II–IV) were found among women age 55 years and older (aOR 0.42; 95% CI 0.23–0.78) compared to women under 40 years. Higher odds of being diagnosed at Stage 1 were found among women who had been screened (aOR 1.86; 95% CI 1.13–3.05) compared to women who were not screened, and among women with adenocarcinoma (aOR 1.79; 95% CI 1.03–3.11) compared to women with squamous cell carcinoma.

**TABLE 4:** Unadjusted and Adjusted Logistic Regression Results for Predictors of (a) Screening, (b) Adequacy of Follow ‐up among Screened, and (c) Staging^a^

	a. Screened (vs. Not Screened) (n=376)^b^			b. Adequate (vs. Inadequate) Follow‐up Among Screened (n=122)^c^			c. Stage I (vs. Stages II–IV) (n=349)		
	No.^d^	uOR (95% CI)	aOR (95% CI)^e^	No.d	uOR (95% CI)	aOR (95% CI)^e^	No.d	uOR (95% CI)	aOR (95% CI)^e^
Age at Diagnosis, y									
23–39	113	Reference	Reference	58	Reference		103	Reference	Reference
40–54	156	**0.39 (0.24–0.64)**	**0.43 (0.25–0.73)**	44	1.18 (0.52–2.68)		146	**0.57 (0.34–0.97)**	0.74 (0.43–1.29)
≥55	107	**0.24 (0.14–0.43)**	**0.26 (0.14–0.47)**	20	3.46 (0.91–13.19)		100	**0.36 (0.20–0.63)**	**0.42 (0.23–0.78)**
Race/ethnicity									
Non‐Hispanic white	249	Reference	Reference	88	Reference		229	Reference	Reference
Other	127	**0.54 (0.34–0.85)**	0.96 (0.56–1.66)	34	0.72 (0.31–1.63)		120	0.72 (0.46–1.13)	
Income									
< $30,000	163	Reference	Reference	40	Reference	Reference	155	Reference	
$30,000–89,999	114	**0.54 (0.34–0.85)**	1.45 (0.82–2.58)	38	2.37 (0.93–6.00)	2.09 (0.75–5.84)	104	1.53 (0.93–2.51)	1.23 (0.71–2.14)
≥ $90,000	67	**5.74 (3.11–10.58)**	**3.62 (1.76–7.43)**	38	**4.18 (1.51–11.58)**	**3.96 (1.24–12.64)**	61	**2.20 (1.19–4.06)**	1.14 (0.56–2.31)
Insurance status									
Not insured	97	Reference	Reference	20	Reference	Reference	94	Reference	Reference
Insured	273	**2.97 (1.74–5.08)**	**2.09 (1.15–3.81)**	100	**3.16 (1.19–8.39)**	1.77 (0.54–5.73)	249	**1.74 (1.08–2.80)**	1.33 (0.77–2.29)
Tubal Ligation									
Not done	275	Reference	Reference						
Done	101	**0.41 (0.24–0.68)**	**0.55 (0.31‐0.96)**						
Recommended follow‐up^f^									
Repeat testing				33	**2.93 (1.10‐7.79)**	2.13 (0.74‐6.13)			
Biopsy				42	Reference	Reference			
Biopsy+				47	**4.64 (1.80‐11.96)**	**5.02 (1.85‐13.62)**			
Histology									
SqCa							250	Reference	Reference
Adenocarcinoma							93	**2.10 (1.27‐3.46)**	**1.79 (1.03‐3.11)**
Other cancer							6	5.00 (0.58‐43.41)	2.88 (0.32‐25.93)
Screening status^b^									
Not screened							213	Reference	Reference
Screened							136	**2.58 (1.64‐4.05)**	**1.86 (1.13‐3.05)**

Abbreviations: 95% CI, 95% confidence interval; aOR, adjusted odds ratio; SqCa, squamous cell carcinoma; uOR, unadjusted odds ratio.

^a^ Multiple imputation of missing data was conducted for use in logistic regression models for all three outcomes.

^b^ Women were classified as having been screened for cervical cancer if they had at least one screening test (Pap, with or witho ut HPV) 6 months or more before the date of diagnosis.

^c^ Follow‐up was among screened women with at least one abnormal screening test result.

^d^ Counts represent distributions prior to multiple imputation for missing data.

^e^ Adjusted models include variables found to be significant at p < 0.05 in unadjusted analysis.

^f^ Recommended follow‐up after the first abnormal Pap or positive HPV test recorded in the 5‐year study period among women with at least one abnormal test was grouped into three categories according to recommended follow‐up: repeat testing or optional colposcopy (“repeat testing”); colposcopy within 3 months (“biopsy”); possible high grade finding and/or excisional procedure recommended (“biopsy+”).

## 4 DISCUSSION

Cervical cancer is largely preventable with appropriate screening and follow‐up, as precancerous lesions can be found prior to invasion, yet women continue to develop this disease. This unique population‐based study using cancer registry data to capture women with cervical cancer and chart data to verify medical history prior to diagnosis confirms that most women with cervical cancer have not been adequately screened or followed. Sixty percent of women in this study had not been screened. The use of existing or development of new tailored interventions among unscreened women to increase screening could have the greatest potential in decreasing the burden of this disease.

This study provides data that could be used to highlight areas of improvement at all levels of care including: (a) federal programs that increase cervical cancer screening, (b) state and local public health organizations to encourage women to get screened by working with state Medicaid programs, community health centers, and community‐based groups, (c) medical practices to integrate a discussion on cervical cancer screening history into the clinic workflow, and (d) education for women to learn about screening options and follow‐up for abnormal results.

Findings from this population‐based study are comparable to those from similar studies conducted in managed care health plan setting, a safety net system and state‐wide evaluation that found proportions of women without screening of about 50%, 60%, and 64%, respectively.^6^, ^7^, ^11^ Like similar studies, this study found that screened women had higher incomes and were more likely to be insured than unscreened women. Significant disparities in cervical cancer screening exist for women who live in poverty, have limited access to care, or are racial/ethnic minorities.^1^, ^15^, ^16^, ^17^

The CDC through the National Breast and Cervical Cancer Early Detection Program (NBCCEDP) funds states, territories, and tribal organizations to help low‐income, uninsured, and underinsured women gain access to cervical cancer screening, diagnostic testing, and referral to treatment.^18^ However, the NBCCEDP is able to serve only a small portion (approximately 7%) of women eligible for the program.^19^ Other national programs also provide cervical cancer screening to uninsured women; however, insurance‐related disparities in screening have been observed even though screening is provided in those programs regardless of insurance status.^20^

The distributions of cervical cancer histologies by screening status and screening test in this study were like findings from previous studies.^5^, ^6^, ^7^, ^11^ It has been suggested that the proportion of new squamous cell carcinoma cases is likely to be lower in screened populations because precursors of squamous cell carcinoma are detected more easily by Pap test, the main method of screening.^21^ We found that 70% of women in our study had squamous cell carcinoma; this proportion was higher for unscreened women (78%) than for screened women (57%). Adding HPV testing to Pap testing improves the detection of adenocarcinoma.^22^ Almost 40% of screened women in this study had adenocarcinoma; of these, more than half (56%) had a co‐test 6 months or more before diagnosis, of which 75% were positive for HPV. Adenocarcinomas had about twice the odds of being diagnosed at Stage I compared to squamous cell carcinomas.

Twenty‐six screened women had a history of only normal screenings other than abnormal Pap and HPV results collected on the day of diagnosis. Of these, 20 women had multiple Pap tests throughout the study period and 10 had at least one HPV co‐test. The normal results of these 20 women may reflect false negatives, a limitation of screening tests. Other studies investigating cervical cancer screening, follow‐up, and diagnosis also found screening detection failures.^7^, ^8^, ^9^, ^10^

Not many studies have addressed adherence to follow‐up of women with abnormal screening tests outside of insured populations^11^; the CICC Study found that over one‐third of women with abnormal test results did not receive adequate follow‐up. Women whose test results required a repeat test (lowest urgency) or biopsy with more aggressive procedures in 1–3 months (highest urgency) were most likely to get adequate follow‐up compared to women who needed biopsy in 3 months (moderate urgency). Women for whom recommended follow‐up included the option of a repeat test had ample time for this noninvasive follow‐up (within 1 year) and may have contributed to higher adherence compared to the biopsy group. Additional education on reviewing test results that call for immediate action per management guidelines––like a biopsy––can help providers and patients better understand the need for immediate treatment. Higher income and having insurance were predictors of adequate follow‐up; this suggests that in addition to education, assisting women in covering follow‐up costs may increase timely follow‐up. For women diagnosed with cervical cancer in the NBCCEDP, over 90% receive diagnostic care within 90 days, and the median time to treatment referral is about 21 days.^23^ Lessons learned from the NBCCEDP can provide insight into how to improve follow‐up and treatment in other US settings.

Findings from this study can also be used to encourage providers to use every opportunity to screen, especially if the patient had a tubal ligation procedure. This study found that women with a tubal ligation were about half as likely to be screened and compared to those without the procedure.

Other studies have also found that women with tubal ligation were less likely to be screened, suggesting that not having to visit a provider for contraception may decrease opportunity for screening.^24^, ^25^

This study had some limitations. We cannot be sure that we obtained all medical records for the entire 5 years prior to diagnosis. However, the chart abstraction protocol included a process for assessing the completeness of the medical records obtained by the study staff.^4^ Sensitivity analyses were conducted that included only cases where medical records were judged to be complete. We tested the potential impact of missing data due to failure to obtain complete medical records by excluding the 125 observations with suspected incomplete data and re‐testing all logistic models. No statistically significant differences in findings were observed. We chose a 6‐month cutoff for screening, assuming any such testing performed within 6 months of diagnosis could have been for diagnostic purposes, an assumption used in other studies.^5^, ^7^, ^11^ In determining the definition for screening at 6 months, we examined other thresholds for defining screening (4 months and 8 months), but these changed the rate of screening only minimally (44% and 38%, respectively, vs. 40% at 6 months) and did not affect other findings. In addition, because this was a study of cervical cancer survivors, not including women who were deceased could bias our results to underreport the unscreened estimate. In the methods paper, the enrolled cervical cancer survivors were compared to those that were deceased during the study period. As expected, deceased women had later stage disease and were older (>65 years) compared to enrolled women.^4^ Additionally, follow‐up was based on the follow‐up to only the first abnormal test. Finally, with a sample from only three states this study may not be generalizable to the United States overall. However, demographic characteristics (age, race, and ethnicity) of women enrolled in this study were similar to those of women in the US diagnosed with cervical cancer.^4^

An important risk factor for cervical cancer is not being screened. This study found that 60% of enrolled cervical cancer survivors did not receive appropriate screening in the 5 years prior to their diagnoses. Increasing screening in rarely and never‐screened women, as well as, timely follow‐up is crucial to continuing to lower cervical cancer incidence in the United States.

## DISCLOSURE

The findings and conclusions of this manuscript are those of the authors and do not necessarily represent the official position of the Centers for Disease Control and Prevention.

## AUTHOR CONTRIBUTIONS

VBB led the writing of manuscript. JEJ and AG conducted data analysis and contributed to writing of the manuscript; WKH contributed to methodology; VS, CCT, and LCR contributed to the writing of manuscript draft. All co‐authors reviewed and edited the manuscript.

## Data Availability

The data that support the findings of this study are available on request from the corresponding author. The data are not publicly available due to privacy or ethical restrictions.
